# Cognitive testing of questions about antenatal care and nutrition interventions in southern Nepal

**DOI:** 10.1016/j.socscimed.2022.115318

**Published:** 2022-10

**Authors:** Andrew L-Thorne-Lyman, Tsering Pema Lama, Rebecca A. Heidkamp, Porcia Manandhar, Seema Subedi, Melinda K. Munos, Emily Bryce, Subarna K. Khatry, Steven C. LeClerq, Joanne Katz

**Affiliations:** a Department of International Health, 615 N. Wolfe Street E2545, Johns Hopkins Bloomberg School of Public Health, Baltimore, MD, 21205, USA; b Nepal Nutrition Intervention Project-Sarlahi, Kathmandu, Nepal

**Keywords:** Nepal, Survey, Nutrition, Iron, Antenatal care, Cognitive, Recall, Validity

## Abstract

Large scale surveys such as the Demographic and Health Surveys (DHS) are used to measure the coverage and quality of antenatal care (ANC)-related services. Studies have increasingly validated questions from these surveys, though few have explored respondent comprehension or associated thought processes. This study aimed to use cognitive testing and validation approaches to understand how survey respondents understand questions related to ANC-related nutrition services. The study was nested within a larger validation study in southern Nepal. Pregnant women's receipt of ANC related services was directly observed at five health posts followed by a recall interview at 6 months postpartum. A week later, a survey module was re-administered to 30 women containing 15 questions about receipt of ANC care and specifically nutrition-related services. Detailed probing was used to identify cognitive challenges related to comprehension, retrieval, judgement, and response. Respondents accurately recalled the four specific ANC visits recommended by the government of Nepal but those with more visits struggled to estimate the total number of ANC visits they had made. A number of terms including “antenatal care, “nutrition” and “breastfeeding” were challenging for many respondents to understand. Visits to private providers including for ultrasounds were inconsistently included in ANC visit counts suggesting that question wording could better specify the type of care. Many respondents over-estimated the number of iron folic acid (IFA) supplements taken during pregnancy, and recall was challenging. Calculations were based on estimating the number of months between first ANC visit to delivery, and only sometimes factored in missed tablets. Opportunities exist to improve questions to facilitate better comprehension by respondents through a combination of using local terms and explanations, reordering some questions, and adapting questions to better match respondents' approaches to estimating numeric responses.

## Highlights

• Cognitive interviewing identified problems with understanding of survey questions.
• Words for ANC, breastfeeding and nutrition were not well understood by all women.
• Women accurately recalled visits from ANC cards but total visit estimates were hard.
• Overestimates of iron supplements consumed may result from estimation processes used.

## 1 Introduction

Health systems in many low and middle income countries (LMIC) implement nutrition interventions during pregnancy recommended by the World Health Organization including micronutrient supplementation, dietary counseling, weight gain measurement, and breastfeeding counseling (World Health Organization (WHO), 2016; WHO, 2018; WHO, 2020). However, estimating coverage, defined as the number of people receiving services by the total number of people who should receive services in a population, has been challenging. Data routinely collected alongside delivery of antenatal care (ANC) is useful for monitoring but is often not sufficiently representative or of sufficient quality to provide accurate population-based estimates of intervention coverage.

Many countries rely on large-scale household and facility surveys to gather information about the coverage and quality of ANC. Demographic and Health Surveys (DHS) are nationally representative, large-scale household surveys that are designed to collect harmonized data on a variety of topics in more than 90 LMIC. The DHS core questionnaire includes a number of specific questions related to the receipt of nutrition-related services. The DHS-7 version of the questionnaire posed these questions to women that had delivered a live birth in the previous five years and in DHS-8 this was changed to women with live or still births in the previous three years (DHS Model Questionnaire - Phase 8, 2021).

Validating the questions that are used to measure coverage in large scale has been an area of growing research. Recent studies have validated questions related to topics of interest including questions related to family planning service quality, birthweight, delivery practices, breastfeeding, iron folic acid coverage and nutrition counseling (Blanc et al., 2016; Ameen et al., 2021; [Bibr bib17][Bibr bib33], Bryce et al., 2022). Yet the reasons why certain questions may lead to inaccurate estimates of coverage in different settings have not been as well explored. Mismatches between the intent that survey developers have when developing questions vs. the interpretations of respondents may occur, are likely to vary across settings, and are sometimes addressed through pretesting, but have seldom been explored through research (Ashok et al., 2021).

Cognitive interviewing is a qualitative research method used to understand the processes through which respondents listen to, process and respond to survey questions (Willis, 2004). Various approaches including probing are used to evaluate whether the survey questions are generating the intended information from respondents and to identify cognitive challenges impairing comprehension (Beatty and Willis, 2007). Cognitive interviewing can help to identify strategies to improve comprehension and accuracy of responses including changes in wording or framing (Willis, 2004; Beatty and Willis, 2007). There is a common set of cognitive challenges frequently identified in survey questions including 1) poor comprehension (such as what respondents believe a question is asking and what they understand to be the meaning of specific words and phrases) (Willis, 2004), 2) difficult retrieval of information from memory particularly for questions that involve estimation or recall of the number of events, 3) retention of multiple concepts presented in a single question and 4) confusion about the time period being referred to (Willis, 2004).

The present study is part of a wider research effort in the southern plains of rural Nepal to validate and test household survey questions related to ANC that are either currently used in large-scale surveys or are in development (Bryce et al., 2021, Bryce et al., 2022). Prior studies published from this work found that women were unable to accurately recall the number of IFA tablets received during pregnancy, resulting in inflated tablet count estimates, and that women were less likely to recall receipt of nutrition counseling accurately compared to a more tangible, physical intervention like blood pressure measurement (Bryce et al., 2021, Bryce et al., 2022). However, the reasons for lack of accuracy remain unclear.

This study has two main aims: 1) To understand how survey participants comprehend questions related to antenatal nutrition services, and 2) To explore factors that may affect women's ability to give accurate responses. These aims inform the ultimate objective of trying to identify potential ways to improve how questions are posed to improve the accuracy and quality of responses.

## 2 Methods

### 2.1 Study setting

This study took place at the Nepal Nutrition Intervention Project Sarlahi (NNIPS) field site in Sarlahi on the southern plains (*Tarai)* of Nepal. The study population is largely rural agrarian and culturally and linguistically heterogeneous and includes both Nepali speaking and Maithili speaking groups (Mullany et al., 2008).

Province 2, where Sarlahi is located, has Nepal's highest prevalence of adolescent pregnancy and malnutrition in women of reproductive age (Ministry of Health and Nepal; New ERA; and ICF, 2017). In 2016, Province 2 had the lowest rates of ANC coverage in the country despite having the greatest physical proximity to health services: 70% of households lived within 30 min of a government health facility. Nepal continues to follow the WHO's 2002–2016 focused ANC model (FANC) with visits in the 4th, 6th, 8th^,^ and 9th months of pregnancy and clearly defined services delivered each of these time points. (WHO, 2002). In 2016 only 36.1% of women in Province 2 received all four recommended visits compared to 58.8% nationally (Ministry of Health and Nepal; New ERA; and ICF, 2017). Province 2 has the lowest rates of women's education in the country and ANC attendance has been positively associated with education in previous studies (Adhikari et al., 2020).

Nutrition interventions delivered through ANC in Nepal include iron folic acid supplements, checking of weight, and counseling on nutrition and breastfeeding. Nepal incentivizes ANC participation by providing 800 rupees (∼7 USD) at the end of pregnancy to women who turn in an ANC card with each of the four required visits marked. (Witter et al., 2011). These four visits are the only visits recorded on the card.

### 2.2 Study design and implementation

**Fig. 1:**
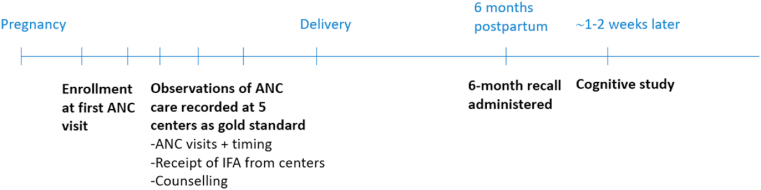
Timeline of data collection for the cognitive study, 6-month recall and observations.

Fig. 1 illustrates the study's several rounds of data collection. The cognitive study was nested within a validation study described in detail elsewhere (Bryce et al., 2021, Bryce et al., 2022). In brief, study staff directly observed and recorded receipt of ANC services by enrolled women (Munos et al., 2018) at five government health posts using a 28-item checklist. The checklist included receipt of iron folic acid pills, whether the woman was instructed to buy iron folic acid tablets or syrup, measurement of weight, counseling on nutrition and breastfeeding, and other non-nutrition services not explored in the present study. At each subsequent ANC visit following the enrollment, women were asked whether they had sought any care at non-study clinical sites. They were also asked about receipt or purchase of IFA as well as counseling from other sources. Approximately 6 months after delivery, women in the validation study were visited at their homes or parental homes, and asked about ANC services received using a questionnaire, described in greater detail below.

### 2.3 Inclusion criteria

The validation study included pregnant, married women age 15 years or older, living in the NNIPS study area who attended their first ANC visit at one of the five health posts in the study area and expressed intention to return to these study sites for all subsequent ANC. From the 441 women enrolled in the validation study we purposively selected 30 women ≥18 years of age to participate in the cognitive interviewing study. The sample was drawn to include about a 50/50 mix of participants in regard to parity (none vs. 1+ prior pregnancies) and language spoken (Nepali vs. Maithili) as it was felt that these characteristics may influence responses and use of services.

### 2.4 Questionnaire content and translation

Data on basic sociodemographic factors was collected during the first validation study visit. The 6-month validation study visit questionnaire included additional questions about socioeconomic status and the receipt of ANC services at the study health posts. The origin of these questions came from the official translated Nepali DHS 2016 questionnaire (DHS Round 7), a draft of the DHS Round 8 questionnaire (DHS Model Questionnaire - Phase 8, 2021) and additional questions which we developed to assess receipt of information and counseling related to nutrition and breastfeeding and weight gain assessment developed using approaches outlined for infant feeding counseling (Choufani et al., 2020) (Supplementary Table 1**)**. These questions were translated and backtranslated by Nepali/English speakers familiar with the study context. Interviewers fluent in both Maithili and Nepali conducted the interviews in Maithili using a standardized approach.

### 2.5 Cognitive interviews

Four interviewers with experience conducting surveys and qualitative research in this setting carried out the cognitive interviews. They participated in a week-long training on the interview guide including probing and standardized notetaking methods. The interview guide was revised based on field testing with validation study participants who were ineligible for the cognitive study. All cognitive interviews were digitally recorded, and interviewers took field notes.

The cognitive interviews were conducted between September and December 2020, approximately a week after each participant's 6-month interview for the validation study. Following brief explanation of the research approach, the household survey question module (Supplementary Table 1) was administered with the goal of replicating the style of a DHS interview. As participants responded to questions, interviewers identified and recorded verbal and non-verbal cues such as taking an abnormally long or short time to reply to a question, pauses, changes in voice tone, asking for repetition or clarification of the meaning of words and facial expressions potentially indicating confusion.

The administration of the survey module was followed by qualitative cognitive interviews (Beatty and Willis, 2007). A probing method for cognitive interviewing was chosen over a “think aloud” approach based on positive experiences with the probing technique from a similar study in India (Ashok et al., 2021) and because it was felt that interview-directed probing would be more familiar to respondents than verbalizing their thought process. Interviewers were trained to probe more deeply on the questions with documented cues, as described above. Probes informed by cognitive stages were used to explore issues of comprehension, response, retrieval and judgement (Ashok et al., 2021).

Precautions taken to prevent the spread of COVID-19 included masking by interviewers and respondents, handwashing with soap was provided to interviewees, holding interviews outdoors when weather permitted, and maintaining a distance of >6 feet during data collection. Interviews lasted about an hour in total.

### 2.6 Data analysis

Responses to the quantitative portions of the cognitive study were entered into a REDCap server database and compared to the 6-month validation study interview results. Cognitive interviews in Maithili were translated into Nepali and reviewed by a team of Nepali speaking researchers and all interviews were listened to directly by a team of three Nepali speaking researchers. For analysis, a spreadsheet was created listing each survey question, responses, and notes from the interview about potential problems. As researchers listened to the interviews, they summarized findings in the spreadsheet, extracting verbatim text of responses where rich explanation was provided. Data were analyzed by examining frequency of difficulty for each question across respondents and to identify patterns of the type of problem experienced overall and by respondent characteristics.

For questions involving estimation (i.e., number of iron folic acid tablets consumed and number of ANC visits), descriptive statistics were used to compare data from each cognitive study participant's observed ANC visits, and 6th month validation study visit with her responses to the same questions during the cognitive interviews. Stata IC16 was used for all quantitative analyses and to generate figures.

### 2.7 IRB approval

This study, including permission to collect/resume data collection during the COVID-19 epidemic, was approved by the institutional review board at Johns Hopkins Bloomberg School of Public Health and by the Nepal Health Research Council. Signed consent was obtained for the parent validation study and verbal consent prior to the cognitive interview.

## 3 Results

Average age of the cognitive testing study participants (n = 30) was 22 years (Table 1). Half of the interviews were conducted in each of the two languages (Nepali/Maithali) and half of the women were primiparous. Formal education levels were about evenly distributed between none, some primary, and 6 level or beyond. About half of the women received at least 5 observed ANC visits during the validation study. Table 2 summarizes key issues identified across the questions and examples of the two major cognitive challenges identified in the study follow:

**Table 1:** Characteristics of participants (n = 30)′.

Characteristic	Percent or mean (sd)
Mean age at baseline (y), mean, (sd)	22.2 (4.1)
Age <20 y old (%)	30
Language of interview (%)	
Nepali	46.7
Maithili	53.3
Number of live births (mean, (sd))	0.77 (0.94)
Primiparous (%)	50.0
Highest education (%)	
0	36.7
1-5	30.0
6+	33.3
# Observed antenatal care visits last pregnancy (%)	
1	10.0
2-3	10.0
4	23.3
5+	56.7

**Table 2:** Nutrition-related questions, their origin and identified problems.

Questions	Problems identified^*a*^			Suggestions for addressing problems
	Unfamiliar words or comprehension	Judgement or estimation	Response options	
*20. How many months pregnant were you when you first received antenatal care for this pregnancy?*	**			- Important to use locally understood words for pregnancy and ANC (applies to multiple questions below) - Define the specific type of visits that should be counted given that care may be provided by both public and private providers.
*21. How many times did you receive antenatal care during this pregnancy?*	**	**		
*21.a. Did you receive antenatal checkups in the following months during this pregnancy? a) when you were* 4 months *pregnant* b) *when you were* 6 months *pregnant* c) *when you were* 8 months *pregnant* d) *when you were* 9 months *pregnant*	**			- The 4 visits were well understood and recalled but re-order 21a in front of 21 given salience of the 4 visits followed by probing about additional visits depending on the definition of ANC care.
*23. During this pregnancy, were you given or did you buy any iron/folic acid tablets?* *[*Show photo of tablets*]*	**	*		- Test alternative wording for ‘during this pregnancy’ including ‘*the time that the baby was in the stomach*’ and ‘*the* 9 months *of pregnancy*’
*23.a. During the whole pregnancy, for how many days did you take the tablets?* If answer is not numeric, probe for approximate number of days		***	*	- Alternative approaches to estimation should be explored including step by step processes starting with months or strips tablets were taken - Reconsider whether # days may be an unrealistic degree of precision to ask for given long recall period
*23b. Where did you get these tablets?*				- Well understood
*26. During this pregnancy, at any antenatal visit, were you weighed?*	* *			- Alternative words for “weighed” may be useful such as asking “did they tell you how many kilograms you were?”
*26.a. During your entire pregnancy, were you weighed once or more than once?*	**		**	- Response options not intuitive for women. Could reword about number of times women were weighed and code accordingly.
*26.b. During this pregnancy, at any antenatal visit, were you told about your weight, weight gain or weight loss?*	**			- Too many parts to this question. Separate probing questions could be asked (1) were you told about your weight (2) were you told how much you had gained or lost?
*27. During this pregnancy, at any antenatal visit, did you receive any information about nutrition or diet?*	**			- The word for nutrition (*poshan*) is a formal translation of the word “nutrition” and was not well understood in this population. - Not well understood. Asking about specific messages given may be better understood. - Most women volunteered information about the messages they received when asked this question. Reordering the questions so that 27b comes before 27a would allow for a better flow of information.
*27.a. How was the information shared?* First allow respondent to provide any answers and check all that apply. Then probe to identify each response and record all mentioned [ask each answer individually]	***		**	- Closed/focused probing more likely to capture many of the channels of information sharing though possibly subject to social desirability bias
*27.b. What information or messages did you receive during your pregnancy about nutrition or diet?* First allow respondents to provide any answers and check all that apply. Then probe to identify each response and record all mentioned [ask each answer individually]	*		**	- Closed/focused probing more likely to capture many types of information/messages
*32. During this pregnancy, at any antenatal visit in the clinic, did you receive any advice about breastfeeding?*	***			- Reword breastfeeding (*stanpnpann)* to “feeding mothers milk” for each of these questions to enhance comprehension
*32.a. During this pregnancy, at any antenatal visit in the clinic, did you receive any information about breastfeeding your baby as soon after birth as possible?*	*			
*32.b. During this pregnancy, at any antenatal visit in the clinic, did you receive any information about breastfeeding your baby exclusively for at least* 6 months*?*	*			

a Asterisks signify extent of difficulties identified during the interview.

*** = Problematic, affecting majority of respondents.

**Semi problematic.

* = minor problem.

### 3.1 Cognitive issue #1: unfamiliar words or concepts

The most common issue identified across questions was the use of unfamiliar words and translation issues.

#### 3.1.1 Difficulties understanding the terms for pregnancy and antenatal care

One issue affecting multiple questions was that the term commonly used for pregnancy (*garbawastha*) in Nepali was not immediately understood and required explanation the first time it was used. This was true for both Nepali and Maithili speaking women, particularly younger women with low education.

“*If you say like that (do you have a baby in your stomach) around here then they will understand at once, the other way (using the term garbawastha for pregnant) won’t be understood”* -Maithili speaking women, age 18, primary school education

***Interviewer*** (I)***:****When you first received your ANC, how many months pregnant were you?*

***Respondent (R):****(long pause, and then faintly says) 4*

***I:****Bahini (sister), were you able to understand [the question]? In this pregnancy, the first time you had an ANC visit, how many months pregnant were you?*

***R:****4 [months] … check-up. I did not understand [smiles shyly]. Can you say again?*

***I:****In this pregnancy, the first time you went for ANC, how many months were you pregnant? In your stomach, how many months was the baby in your stomach when you first went for examination?*

***R:****4 [months], back then it was 4*

-Nepali speaking woman, age 21, no education

Similar confusion was apparent around the words used to describe antenatal care in Nepali, “*garbhawati jaanch*” (pregnancy examination).

***I:****What is garbhawati jaanch?*

***R:****When we have a baby in the stomach it is the examination done at that time*

***I:****Do you think other women will understand this term?*

***R:****They would if you ask, “Pet ma bacha huda kati patak jachauna gayo?” (How many times did you go for check-up when you had a baby in your stomach)*

Maithili speaking woman, age 22, some primary education

The term for examination was understood by most women, yet there was lack of certainty around what type of services were included, around particularly sonograms, which are received from private providers in Nepal. Probing of question 21 asking women to count their ANC visits also revealed that women did not usually count the initial clinic visit (in which urine tests were done to confirm pregnancy and screen for asymptomatic bacteriuria) as ANC, particularly when it occurred earlier than 4 months, but rather defined ANC visits as those in which specific services were received including blood pressure monitoring, weighing, inspection of the belly, distribution of iron tablets and for where there is documentation on an ANC card. Potential solutions to these comprehension issues include training survey enumerators to use descriptive phrases about the time when women have a baby in their stomach, referring to visits with specific descriptions of services provided at a visit, and ensuring that translations into local languages are piloted for comprehension.

#### 3.1.2 Confusion about the phrase “during this pregnancy”

The phrase “during this pregnancy” was included in most questions in this module (Table 2) and elicited confusion for some women. A couple of Nepali-speaking women suggested that using the term “*bacha pet ma bhakyeko bela*” (time that the baby is in the stomach) or “*the* 9 months *of pregnancy*” might be more easily understood. In Maithili there is no specific term for pregnancy and interviewers successfully used these approaches to explain pregnancy.

#### 3.1.3 Confusion about the term for breastfeeding

Women had great difficulty understanding the phrase “advice about breastfeeding” due in part to the lack of a specific term for breastfeeding in Maithili. More than 2/3 of women had difficulty with the question and many of the Nepali speaking respondents also did not understand the word “*stanpann*” (breastfeeding). Further discussion with respondents suggested that the words “*ama ko dudh khwanu*” or “to feed mother's milk” would be a better way of describing breastfeeding in this context. Some women reversed their response from “no” to “yes” once the term was further explained. There were some difficulties understanding the latter part of question 32a referring to breastfeeding “as soon after birth as possible”. One respondent who said in response to question 32 that she had started breastfeeding 5 min after birth did not understand the reference to “as soon after birth as possible” when asked question 32a. Questions 32a and 32b about immediate and exclusive breastfeeding were generally well received and understood correctly although four women thought the question about breastfeeding as soon as possible after birth was related to skin-to-skin contact, perhaps due to local messaging related to kangaroo care.

#### 3.1.4 Confusion about terminology in questions about weighing

Most women instantaneously responded to the question about having their weight taken and appeared to understand the meaning of the question but some women suggested that alternative words could be more easily understood in the community. As explained by one woman, “*Educated women will understand both “wajan” and “taul”. But uneducated women will better understand “jokhyo”.* Another woman suggested that the words “kg” or “kilo” could be used for Maithili speaking women.

Many respondents asked that the next question about frequency of weighing (Question 26a) be repeated and most answered with the actual number of times they were weighed. As one woman explained, “*in my opinion, it is how many times weight was taken and it's about the machine, sometimes more happens, sometimes less and it can be one time or two times too.”* It seems that simplifying the question to ask about the number of times women were weighed, with categorization by the enumerator or during analysis, could help prevent confusion.

#### 3.1.5 Confusion around nutrition counseling terms and responses

Women expressed confusion about questions and response options related to the questions we had developed around receipt of counseling on nutrition and diet, as well as sources of information and messages received. Some women expressed concern that others might not be familiar with the word for nutrition (*poshan*), which is a very formal word for nutrition in Nepali, and not familiar to Maithli speakers. Adjusting the question to reflect specific messages given as part of counseling, such as asking about whether they had been told about “*tagitilo khana*” or strength giving foods, could be a way of enhancing women's recall of counseling.

Women were then asked a series of questions about the way information was shared and then what messages they had received (Table 3). These were difficult questions for many women to initially understand. Repetition and extensive probing were necessary. Separate codes were used for open-ended vs. probed recall, allowing us to explore how salient different responses may be in women's minds.

**Table 3:** Responses to questions about the specific type nutrition counseling information and elements received (n = 29).

	Not received (n)	Received no prompting (n)	Received with prompting (n)
*How was the information shared?*			
Group counseling	13	1	15
One-on-one counseling	6	10	13
Paper or booklet to take home	25	0	4
Poster/sign on wall	10	5	14
Other^a^	27	1	
*What information or messages did you receive during your pregnancy about nutrition or diet?, % (n)*			
Eat more (quantity)	3.5 (1)	34.5 (10)	62.1 (18)
Eat a variety of nutrient rich foods		96.6 (28)	3.5 (1)
Take iron tablets (IFAs)		3.5 (1)	96.6 (28)
Take calcium tablets	20.7 (6)	3.5 (1)	75.9 (22)
How to manage nausea/vomiting	51.7 (15)	3.5 (1)	44.8 (13)
Other (specify)	93.1 (27)	3.5 (1)	3.5 (1)

a One “not sure” for other.

For the ‘how was information shared” question, one-on-one counseling was reported without any prompting by about a third of women, and more women affirmed this when prompted, suggesting it was understood. Several women tended to first answer by describing the key messages they received rather the way they were communicated. It was also unclear whether “information shared” was a domain that women associated with written materials. One woman confused the “paper or booklet to be taken home” with the ANC check-up card.

For the next question, which asked women to recall the messages they received, the response categories related to food intake were salient and needed less prompting than messages about iron and calcium tablets. However, most women were able to recall that they had received messages about supplements after prompting (Table 3). When probed about what she had been told, one woman explained, “*drink milk, eat ghee, meat, fish, and all … spinach and vegetables … should eat roti, rice and all … eat on time, eat throughout the day 3-4 times*.” Given that most women tended to discuss the specific messages that they had received as soon as food related issues were raised in questioning, it seems it would be helpful to reorder the questionnaire so that questions about the content of messages would be asked before mode of sharing.

### 3.2 Cognitive issue #2: judgement/estimation

A number of challenges were identified related to recalling the frequency of ANC use and iron supplementation during pregnancy, and cognitive interviewing allowed us to explore the processes that women used in generating their estimates.

#### 3.2.1 Estimation of the timing of first ANC visit

**Fig. 2:**
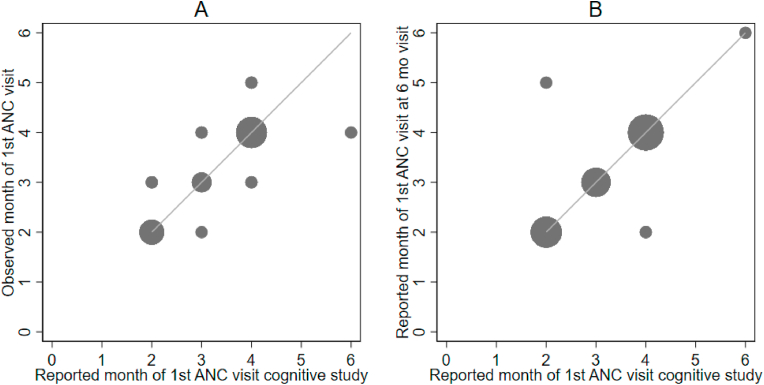
Reported month of first ANC visit vs. observed gestational month of first ANC visit. Size of each data point represents number of values at that data point.

When asked to estimate how many months pregnant they were when they first received ANC (Question 20), women's responses ranged from 2 to 4 months of pregnancy. When compared to validation study data, 96% of cognitive study participant's responses were accurate within a month of the actual visit and 62% recalled the same month as observed (Fig. 2). Women were also consistent in their responses over time, 93% of women gave the same response at the 6-month validation study visit and the cognitive study interview 1–2 weeks later.

It is important to note again that during the validation study, women self-reported visits to non-study sites. A woman's response to whether or not she got ANC services elsewhere depended on whether she personally perceived a service to be ANC. Similar to the discussion of cognitive issue 1, when probed, a few women described early clinic visits that they did not appear to include in their estimate of services received (e.g., visits for pregnancy tests prior to 4 months of gestational age). Others were less consistent, such as the visit described below by a woman who reported her first visit being at 4 months during her cognitive interview but at 2 months in her 6-month interview a week or two earlier:

***I:****Before I asked you about the first time you sought pregnancy examination for this pregnancy …*

***R:****4 months*

***I:****Could you talk about how you became sure that you were 4 months in?*

***R:****At 1–2 months, went to check [ANC checkup]. When I went to get checked, they [the clinic nurses] said not now. [They said] when you are 4 months, then come and at 4 months, they’ll make the [ANC] card. If I am in early days, they won’t make the card … either the menstruation stopped or when you reach 4 months they said and once you reach 4 months the card is made. After 4 months the examination starts.*

**−**20-year-old Maithili speaking woman, some primary education

Responses across cognitive study participants suggest women consider both when the ANC card was made and cessation of menstruation in their cognitive processes to identify timing of first ANC visit. As one woman explained “*This is something that women should remember, they should know when their periods stop. So, they would [be able to] say easily*”. In Nepal the ANC card plays a dual role of tracking visits and serving as proof to collect a government financial incentive at the end of the pregnancy. Most women turned in the card to get their cash incentive before the interview, and so no longer had it to directly refer to. More than 40% of women reported that their first visit occurred at 4 months gestation, which also corresponded to the time that cards are made, and the first incentivized visit is recorded. This may have also contributed to the accurate recalls in response to this question.

#### 3.2.2 Estimation of the number of ANC visits

**Fig. 3:**
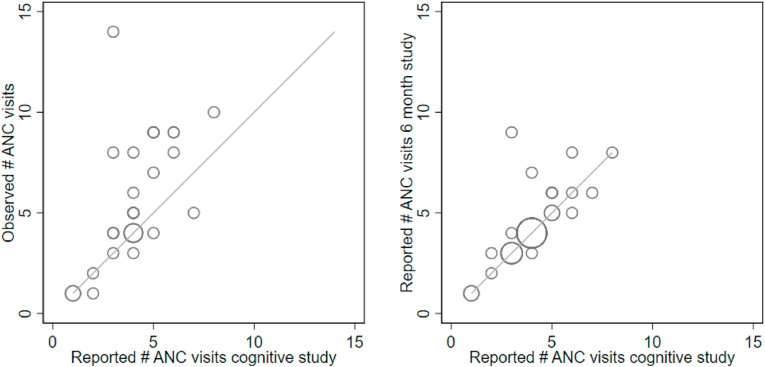
**Alignment between the number of reported ANC visits in the cognitive study vs. observed visits**^1^**(A) and the 6-month recall (B)**^**2**^. ^1^Visits observed in realtime during pregnancy at 4 government health. ^2^Cognitive study data was collected about a week after the 6-month recall.

Women's responses to the recalled total number of ANC visits in the cognitive study were compared to the validation study observations, and the 6-month interview responses obtained about a week earlier. Seven women (23%) reported the same number of ANC visits in the cognitive interviews as were observed (Fig. 3a) and 17 (57%) of women were within one visit of the observed. All but one of the remaining 13 women underestimated their total number of visits compared to observed: a tendency most apparent among those with five or more observed visits (Supplementary Fig. 1**).** Over half of the women (63%, n = 19) recalled the same estimate of ANC visits at both the 6-month and the cognitive interviews; only 3 (10%) of the sample differed by more than 2 visits suggesting the cognitive processes for recall were consistent for most but not all women (Fig. 3b).

##### 3.2.2.1 Reasons for divergent responses

The cognitive interviews revealed several reasons why women may have underestimated the number of visits in their recalls. First, women inconsistently included visits prior to the 4-month mark when ANC cards are commonly issued in their counts. Second, women with frequent and/or closely spaced visits also had difficulty recalling total visits made:

*“I went to the health facility so many times and so I am not able to keep track of exactly how many times I visited. In some months I even went twice or more. In the 9*^*th*^*month, I fell down and had white discharge. So, I went 2 or 3 times. Besides, my family members are so conscious about it that even if I have some pain or some issue, they asked me to go to the health facility. So, I went many times, but just said 6 visits overall.”*

-Maithili speaking woman, age 18, some primary education, first pregnancy

Another woman reported seeking ANC three times in the cognitive interview, nine times in the 6-month interview, and had 14 visits per observation. When probed about inconsistencies, she said “*I went every month of my pregnancy and maybe even more since I had a vomiting issue*”. Observation records showed a number of frequent visits early in pregnancy, sometimes even multiple visits in the same week.

The most frequently reported number of ANC visits during six-month and cognitive interviews was four (Fig. 3), but in most cases, the number of observed visits was higher. One woman who had five observed visits but reported four visits explained:

“*How I remember going four times for examination (ANC) is that she (referring to the auxiliary Nurse Midwife at the health post) gave one card and in that card, it was written on that. Which month, which day, everything was written there.*”

-Maithili 31-year-old woman, second pregnancy, no formal education

Another point of confusion related to the multiple ANC providers in this setting including government health posts and hospitals, private providers offering all ANC services and clinics for sonogram services only. Women were not consistent in whether they counted services from private providers in their recall responses. In response to question 21, one woman first requested clarification of whether the visits being asked about were “private or here (health post).” After the interviewer clarified it was about ANC generally, the woman answered “six times” which included private provider visits. Her thought process was explained as follows:

***I:****You said that you went to a private facility as well. Do you think that is an ANC visit?*

***R:****I think that is an ANC visit as well. When I went to the private facility, I did a checkup and a video x-ray (sonogram) as well*

***I:****Do you think that video x ray is also an ANC visit?*

***R:****Yes, I think so.*

***I***: *How did you count the 6 visits?*

***R:****I visited the private provider 3 times and 3 times in the government health facility. In the beginning, at the 3*^*rd*^*month, I went to the health facility and also the private provider. Then in the 4*^*th*^*month I went to the health facility. Then in the 6*^*th*^*month again I went to the health facility and again in the 8*^*th*^*month. In the 9*^*th*^*month I am confused about whether I went or not. But I think I went in the 9*^*th*^*month also*.

The woman's observation records showed eight visits to government facilities alone, three of which were in a single week, so she may have not counted visits spaced closely together.

Many women said they considered sonograms to be part of ANC care, but they were not consistent in including them in their total number of ANC visits. One woman had previously reported receiving three sonograms from a private provider during the validation study. However, she did not include them in her estimates for the cognitive study or the 6-month interview. When asked she affirmed that sonograms were part of ANC because they were done *“to see if the baby in the womb is good or not, whether there are any deformities or not”.* Despite this statement, she, and many other women that had sought ultrasounds did not include them in their counts.

#### 3.2.3 Estimation of specific ANC checkups (4th, 6th, 8th^,^ and 9th month visits)

Recalls of the four specific ANC visits were quite accurate when compared against observations (Supplementary Table 2). The first (4-month) and last (9-month) visits had the highest validity while alignment between the observed and recall for the 8-month visit was only 50–60%. These questions were not extensively probed as respondents did not seem to have difficulty answering them.

#### 3.2.4 Estimation of the number of iron folic acid tablets received

**Fig. 4:**
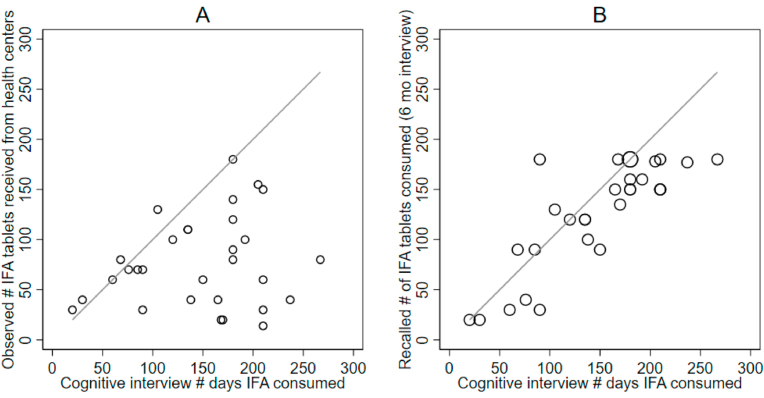
**Alignment between the number of reported iron-folic acid supplements reportedly consumed from cognitive interviews vs received during observed visits**^1^**(A) and reportedly consumed from 6-month recall (B)**^**2**^.

Most women in the cognitive study reported significantly greater numbers of supplements were consumed than the number they were observed to receive at the health center only (Fig. 4a). The mean observed number of supplements received was 79.3, while women reported consuming on average 150 supplements in the cognitive study and 126.6 in the 6-month study. While similar across time, very few respondents gave the same exact answers at both time points (Fig. 4b). Eight of the 30 women reported purchasing iron supplements or syrup in addition to those given through study clinics.

##### 3.2.4.1 How women estimate IFA consumption

Over half of the women recalled values of supplements consumed for both the 6-month interview and the cognitive study that were multiples of 30, this was true for 16/29 women in the cognitive interviews and 20/30 women in the 6-month interviews. Cognitive interviews confirmed that women tend to first think about consumption in terms of months, starting with identifying the gestational age when they first received pills and then reflected on how many subsequent months, they took it. They either converted that number of months into days themselves or required assistance from the interviewer to do so. Many of those who did not answer with multiples of 30 used this same thought process but then factored how many doses they had missed, either themselves or following probes by the interviewer.

Several women seemed intimidated by the level of detail being asked. *“I did not understand the question and I also felt nervous to answer because I did not know what to say*” explained one woman. Another explained “*I felt a bit awkward and scared as well.”* When asked why she felt scared she replied *“you ask about which months I ate [iron tablets] and which months I went [for ANC] … I did not understand and so was afraid*.” She went on to explain, “*I have to remember how many days I did not take* [iron tablets*]. It is a thing from a year ago … how to remember*?“. Other women expressed similar concerns that so much time had passed that it was difficult to recall the specific number of supplements they had taken with the requested precision. As one woman explained “*It has been* 10 months *since for me and I have forgotten some things. Since you are asking these questions, I am slowly remembering some things. Similarly, others may have forgotten and some may remember.*”

##### 3.2.4.2 Suggestions for improvements

Women who were asked about how to improve this question generally supported continuing to ask for the number of days. However, they also affirmed that months are used in the process of estimating and may be the preferred question. As explained by one woman:

***I:****So, you ate these in days. You said in days, and you quickly answered in days. How did you calculate in days? Would it be easier for other women to say in days?*

***R:****not really. I added and told you, other people might not know how to add.*

***I:****In your opinion what would be easier for others to calculate?*

***R****: months, one month, two months*

-Maithili 20-year-old woman, first pregnancy, no formal education

Many women did not account for missed days in their calculations and instead performed a strict conversion from months to days resulting in estimations that clustered at 30-day multiples. However, data from observations showed that in some clinics auxiliary nurse midwives did not provide enough supplements to the woman to last the full period in between visits. Thus, women using the strict approach of multiplying months by 30 but who did not receive or purchase supplements from another source may have overestimated actual receipt or consumption.

One participant said that for her it would be easier to answer in ‘*patta*’ or tablet strips of 10 pills each used by health facilities. Another explained *“People who don't know how to read and write, who are illiterate, people like them should be asked about how many “patta” (strips) they ate, and how they ate it. If asked this way, then it will be easier for them. When asked in days or months, they will not know and will have difficulty*”.

## 4 Discussion

We used a combination of cognitive testing and validation approaches to evaluate whether women in a multi-cultural setting of southern Nepal understand questions about use of nutrition-related ANC services in pregnancy per question intent. Many of the questions were well understood, but we identified some comprehension challenges and opportunities to improve questions. We consider the implications of these findings for the diverse global contexts where similar questions are used as well as for parts of Nepal that may be contextually similar.

Many women did not readily recognize terms used in the Nepali questionnaire including those for pregnancy, antenatal care, nutrition, and breastfeeding. These findings echo similar findings from India, Haiti, and Bangladesh (Ashok et al., 2021; Hannan et al., 2020; Johnson and Diego-Rosell, 2015; Scott et al., 2020). Some of these words did not have a comparable word in the Maithili language and some were not well understood by Nepali speakers either. A challenging element of administering surveys in linguistically diverse contexts like the *Tarai* of Nepal or urban centers across Asia and Africa is that women not only speak different primary languages than those reflected in questionnaire translations but also have varying degrees of familiarity with key words that were important to understanding multiple questions.

A general strategy to help improve comprehension is to pre-test questionnaires in different sub-populations where heterogeneity may exist, and to develop protocols that support enumerators to explain concepts in a way that local populations can understand, an approach that is built into the DHS. In Nepal, the DHS questionnaire is translated into three major languages but administered by enumerators who speak additional languages. Recognizing this challenge, the global DHS manual says “*it is very important not to change the meaning of the question when you rephrase it or interpret it into another language*.” (ICF, 2020) Training enumerators to pick up on verbal and non-verbal cues that signal potential lack of understanding and integrating cognitive interviewing strategies into pre-testing activities during enumerator training could be additional opportunities to identify how to best translate words and concepts to populations that lack a formal translation.

We more specifically explored the thought processes used by women to estimate the number of ANC visits and IFA supplements consumed. Women accurately recalled the four specific ANC visits recorded on ANC cards and required for financial incentives but struggled to accurately estimate the total number of ANC visits. It is not clear whether other contexts are similar in terms of how ANC cards are filled and incentivized but in Nepal these are clearly important anchors for recall. The generalizability of such findings to other contexts where ANC cards are not completed in front of women and/or incentivized requires further exploration. We also found that many women were able to recall details about the services that they had received as part of visits, which provides potential support for efforts to develop indicators related to quality and content of care (Hodgins and D'Agostino, 2014).

In the DHS-8 manual, women are asked to recall antenatal care given by a healthcare provider—this includes at healthcare facilities but also that provided at pregnant women's homes (ICF, 2020). ANC in southern Nepal and much of South Asia (Jo et al., 2019) is provided by a mix of government and private providers including pharmacies and sonogram providers. Accordingly, we observed heterogeneity in women's perceptions of what provider types or services should be included when they were asked about ANC. Women's recalls of ANC were mostly specific to government care, but several women said that sonograms should be counted as well. Similarly, early visits for pregnancy testing prior to the official ANC card being prepared occupied an ambiguous position in women's counts. When viewed from the perspective of indicator and measurement standardization, it is important that questions and their components are understood in the same way across respondents. It may be beneficial to add wording that helps respondent focus their response to visits that meet the desired definition.

Our study suggests that the recording of specific visits on an ANC card, which are aligned with incentives, make the recall of visits to government facilities more salient. Nepal is currently implementing a 4-visit FANC model but there are plans to move towards the WHO 8+ visit protocol. As long as specific visits are incentivized and recorded on a card, it may be useful to place questions about the recorded visits first and subsequently to ask about additional visits to government and/or private providers. For countries implementing the newer WHO 8+ visit protocol, clearly defining what counts as an ANC and ensuring the question is worded in a way that enables participants to comprehend the intent and respond accordingly is essential.

IFA consumption in pregnancy is an important indicator included in both the Global Nutrition Monitoring Framework and the WHO Nutrition Landscape Information System (WHO, 2017, WHO, 2019). Iron folic acid supplementation in pregnancy is provided free of charge as a component of antenatal care in many countries, but coverage and adherence rates have lagged (Sanghvi et al., 2010; Bourassa et al., 2019). Our finding that many women tended to overreport consumption of IFA mirrors the finding from the parent validation study (Bryce et al., 2022) that women overestimated IFA receipt by 45 supplements on average. In our study, women struggled to recall how many supplements were consumed given the amount of time that had passed since their last pregnancy. A common starting point for estimation was counting how many months they had consumed them for and then (often with the enumerator's help) converting to days. Such a process is also described in the local DHS manual for Nepal. Women's inability to recall how many days were missed and/or probing that directly converts from months to days without factoring in missed days may explain some of the overestimation and why “heaping” at 30-day intervals is a common feature of this type of data. Social desirability bias is another possible explanation for overestimation.

Findings related to recall of nutrition-related counseling builds on a body of work validating women's recall of specific components of care delivered as part of ANC in other settings (McCarthy et al., 2020). A previous study in Bangladesh, Cambodia and Kenya found that women tend to have greater accuracy in recalling physical checks such as abdominal examination and blood pressure than counseling related interventions (McCarthy et al., 2020). The authors suggested that counseling may be more difficult to recall than other elements of care unless delivered in a memorable way. Our findings as well as study in India suggest that the terms “nutrition” and “nutrition counseling” in local translations may not be widely understood (Ashok et al., 2021). We found that women cited messages about the need to consume a variety of nutrient-rich foods without prompting but that other counseling messages about supplement use, how to manage nausea and vomiting, and the need to eat more were only recalled after interviewers asked about them directly (i.e., prompted recall). It may be that the question wording “talk to you about nutrition or diet” led women to focus on the diet and not consider supplements as part of the less understood “nutrition” concept. Women may also acquire knowledge about nutrition from a variety of sources including through counseling and may not think of this as a formal exchange or service being provided as part of care.

The relatively small sample size of our study is a limitation given the heterogeneous population. We aimed to enhance the generalizability of our findings within Province 2 of Nepal by purposively sampling women of varying parity and language. However, this area differs from other parts of Nepal, and as such the generalizability of our findings to other parts of the country is uncertain. We also enrolled women that had come in for ANC at government health posts and the generalizability of findings to women that do not seek care from those providers is therefore uncertain.

The questionnaire we used was much shorter than most large scale surveys, and the order, flow, and content of some questions differed from the women's ANC module in the DHS questionnaires. The questionnaire we administered was a compilation of questions from the DHS-7, DHS-8, and other questions that we sought to validate about ANC care. While we tried to maintain a similar question sequence as DHS, our cognitive study did not include an earlier question about where women received antenatal care for this pregnancy. It is possible that asking this question may prime women to better recall the number of visits later. However, the question was asked as part of the 6-month interviews and similar findings in the estimation of the number of ANC visits at these two timepoints suggest that such a priming effect may be minor.

The DHS currently administers the ANC module to women that delivered within the past three years, while our study only administered to women around 6 months postpartum. It is likely that women's ability to recall pregnancy-related events decreases over time and it is important that future studies explore the validity of such questions out to three years, although prior studies have found little erosion of recall for indicators out to 18 months (Blanc et al., 2016; Chang et al., 2018). Additionally, given the cognitive study was administered just 1–2 weeks after the 6-month validation study visit it is possible that some women recalled the previous interview and we overestimated the actual consistency of responses over time. Finally, due to COVID-19 the nature of interactions in our study may have also differed as our time-limited interpersonal interviews took place outside with masks, perhaps in contexts of less privacy, and with less ability to build rapport than normal.

This was one of the first studies to integrate cognitive interviewing into a validation study and to apply cognitive testing post-hoc to questionnaire implementation. A clear advantage of this approach was that we could triangulate findings of the cognitive study with quantitative validation study data and seek explanation for validation outcomes. At the time we designed and implemented the cognitive interviewing protocol, we did not have access to the findings of the 6-month interviews, but these findings could have used the finding to inform selection of questions and probes by interviewers. Future studies, particularly those utilizing digital real-time data collection, could try to build this into study designs.

Survey data are increasingly used to measure intervention coverage and to monitor progress towards nutrition and health objectives in LMICs. The widespread use of these data underscores the importance of ensuring that the underlying questions are well understood and that participant responses reflect actual receipt of services. Cognitive interviewing studies may be a useful tool for finding appropriate wording and ordering of questions to more accurately measure ANC coverage by understanding how women perceive and report on the care they receive. Cognitive interviewing could also play a role in informing the selection of questions in large surveys where limited space is available by helping investigators determine which questions are well understood.
