# Supplementary material for: Cognitive testing of questions about antenatal care and nutrition interventions in southern Nepal
Source: Soc Sci Med. 2022 Oct;311:115318. doi: 10.1016/j.socscimed.2022.115318 (PMC9554791; doi:10.1016/j.socscimed.2022.115318)
Supplement: Multimedia component 1 [file mmc1.docx]

**Supplementary Table 1.** Questions included in the study and their origin

| **Questions in our study** | **Response codes** | **Question Origin*** | **Original survey questions** | **Response codes** |
| --- | --- | --- | --- | --- |
| **Questions asked in the 6 month interview only** | |  |  |  |
| Did you see anyone for antenatal care for this pregnancy? | 0= No  1= Yes  9= Don’t Know | 2016 DHS, **DHS-8** | Did you see anyone for antenatal care during this pregnancy? | Yes….1 No…..2 |
| Whom did you see? Anyone else?  Probe to identify each type of person and record all mentioned  [ask each answer individually] | __ Doctor __Nurse/Midwife/ANM __Health assistant/AHW __Traditional Birth Attendant __FCHV __Other___  0= No 1= Yes 9= Don’t Know | **2016 DHS**, DHS-8 | Whom did you see? Anyone else?  PROBE TO IDENTIFY EACH TYPE OF PERSON AND RECORD ALL | **HEALTH PERSONNEL** DOCTOR….A NURSE/MIDWIFE….B HEALTH ASST./AHW…C MCH WORKER…D VHW…E **OTHER PERSON** TRADITIONAL BIRTH ATTENDANT…F FCHV….G OTHER (SPECIFY)____ X |
| Where did you receive antenatal care for this pregnancy? Anywhere else?  ***Probe to identify the source of care*** | [List of specific clinics, redacted for blinding]  0=No 1=Yes 9=Don't know | 2016 DHS, **DHS-8** | Where did you receive antenatal care for this pregnancy? Anywhere else?  PROBE TO IDENTIFY THE TYPE OF SOURCE  IF UNABLE TO DETERMINE IF PUBLIC, PRIVATE, OR NGO SECTOR, RECORD 'X' AND WRITE THE NAME OF THE PLACE(S). | **HOME** HER HOME….A OTHER HOME…B **PUBLIC SECTOR** GOVERNMENT HOSPITAL…C GOVERNMENT HEALTH CENTER…D GOVERNMENT HEALTH POST…E OTHER PUBLIC SECTOR (SPECIFY)…F **PRIVATE MEDICAL SECTOR** PRIVATE HOSPITAL…G PRIVATE CLINIC…H OTHER PRIVATE MEDICAL SECTOR (SPECIFY)…I **NGO MEDICAL SECTOR** NGO HOSPITAL...J NGO CLINIC...K OTHER NGO MEDICAL SECTOR (SPECIFY)...L OTHER (SPECIFY)...X |
| **Questions below asked at both the cognitive interview and the 6 month interview** | | |  |  |
| 20. How many months pregnant were you when you first received antenatal care for this pregnancy? | ___ ___ 01 to 10= months 99= Don’t know | 2016 DHS, **DHS-8** | How many weeks or months pregnant were you when you first received antenatal care for this pregnancy? | ___ ___ Weeks…1 ___ ___Months…2 998 Don't know |
| 21. How many times did you receive antenatal care during this pregnancy? | Number of times: 01-15 times  99= Don’t know | **2016 DHS, DHS-8** | How many times did you receive antenatal care during this pregnancy | Number of Times ___ ____  Don't know…98 |
| 21.a. Did you receive antenatal checkups in the following months during this pregnancy? | 0= No 1=Yes 9=Don't Know | **2016 DHS** | Did you receive antenatal checkups in the following months during this pregnancy? | YES NO |
| a) when you were 4 months pregnant | ___ |  | a) when you were 4 months pregnant | 1 2 |
| b) when you were 6 months pregnant | ___ |  | b) when you were 6 months pregnant | 1 2 |
| c) when you were 8 months pregnant | ___ |  | c) when you were 8 months pregnant | 1 2 |
| d) when you were 9 months pregnant | ___ |  | d) when you were 9 months pregnant | 1 2 |
| 23. During this pregnancy, were you given or did you buy any iron/folic acid tablets?  Show photo of tablets   *(photo included locally available IFA supplements and syrups)* | 0=No 1=Yes 9=Don't know | **2016 DHS, DHS-8** | During this pregnancy, were you given or did you buy any iron tablets or iron syrup? SHOW TABLETS/SYRUP/MULTIPLE MICRONUTRIENT SUPPLEMENT | Yes….1 No…..2 Don't know…..8 |
| 23.a. During the whole pregnancy, for how many days did you take the tablets?  If answer is not numeric probe for approximate number of days | ___ ___ ___ Days 000-270  999=Don’t know | **2016 DHS, DHS-8** | During the whole pregnancy, for how many days did you take the iron tablets or syrup? IF ANSWER IS NOT NUMERIC PROBE APPROXIMATE NUMBER OF DAYS |  |
| 23b. Where did you get these tablets? | 0=No 1=Yes 9=Don't know  ___ Antenatal visit ___Pharmacy ___Other | **DHS-8** | Where did you get the iron tablets or syrup?  Anywhere else?  PROBE TO IDENTIFY THE SOURCE  IF UNABLE TO DETERMINE IF PUBLIC, PRIVATE, OR NGO SECTOR, RECORD 'X' AND WRITE THE NAME OF THE PLACE(S). | **PUBLIC SECTOR**  (1) GOVERNMENT HOSPITAL . . . . . . . . . . . . . . . . A  (3) GOVERNMENT HEALTH CENTER . . . . . . . B  GOVERNMENT HEALTH POST . . . . . . . . . . C  MOBILE CLINIC . . . . . . . . . . . . . . . . . . . . . . . . D  COMMUNITY HEALTH WORKER/  FIELDWORKER . . . . . . . . . . . . . . . . . . . . . E  OTHER PUBLIC  SECTOR F  (SPECIFY)  **PRIVATE MEDICAL SECTOR**  PRIVATE HOSPITAL . . . . . . . . . . . . . . . . . . . G  PRIVATE CLINIC . . . . . . . . . . . . . . . . . . . . . H  PHARMACY . . . . . . . . . . . . . . . . . . . . . . . . I  PRIVATE DOCTOR . . . . . . . . . . . . . . . . . . . . . J  MOBILE CLINIC . . . . . . . . . . . . . . . . . . . . . . . . K  COMMUNITY HEALTH WORKER/  FIELDWORKER . . . . . . . . . . . . . . . . . . . . . L  OTHER PRIVATE MEDICAL  SECTOR M  (SPECIFY)  **NGO MEDICAL SECTOR**  NGO HOSPITAL . . . . . . . . . . . . . . . . . . . . . . . . N  NGO CLINIC . . . . . . . . . . . . . . . . . . . . . . . . . . . O  OTHER NGO MEDICAL  SECTOR P  (SPECIFY)  **OTHER SOURCE**  SHOP . . . . . . . . . . . . . . . . . . . . . . . . . . . . . . Q  MARKET . . . . . . . . . . . . . . . . . . . . . . . . . . . . . . R  [MASS DISTRIBUTION CAMPAIGN] . . . . . S  OTHER X |
| 26. During this pregnancy, at any antenatal visit, were you weighed? | 0=No 1=Yes 9=Don't know | Original |  |  |
| 26.a. During your entire pregnancy, were you weighed once or more than once? | 1=Once 2=More than once 9=Don't know | Original |  |  |
| 26.b. During this pregnancy, at any antenatal visit, were you told about your weight, weight gain or weight loss? | 0=No 1=Yes 9=Don't know | Original |  |  |
| 27. During this pregnancy, at any antenatal visit, did you receive any information about nutrition or diet? | 0=No 1=Yes 9=Don't know | Original | As a comparison, DHS-8 asks: "As part of your antenatal care during this pregnancy, did a healthcare provider do any of the following: e) Talk with you about which foods you should eat? | Yes….1 No…..2 DK…..8 |
| 27.a. How was the information shared? | 0=No 1=Yes without prompt 2=Yes with prompt 9=Don't know  ___ Group talk at start of the clinic day ___ One to one counseling ___Paper or booklet to take home ___Poster/sign on wall ___Other (specify)___ | Original |  |  |
| 27.b. What information or messages did you receive during your pregnancy about nutrition or diet? | 0=No 1=Yes without prompt 2=Yes with prompt 9=Don't know  ___ Eat more (quantity) ___ Eat a variety of foods/foods rich with iron, vitamin A or C ___ Take iron tablets (IFAs) ___ Take calcium tablets ___ How to manage nausea/vomiting ___Other (specify) ____ | Original |  |  |
| 32. During this pregnancy, at any antenatal visit in the clinic, did you receive any advice about breastfeeding? | 0=No 1=Yes 9=Don't know | Original |  |  |
| 32.a. During this pregnancy, at any antenatal visit in the clinic, did you receive any information about breastfeeding your baby as soon after birth as possible? | 0=No 1=Yes 9=Don't know | Original | As a comparison, DHS-8 asks: "As part of your antenatal care during this pregnancy, did a healthcare provider do any of the following: e) Talk with you about breastfeeding | Yes….1 No…..2 DK…..8 |
| 32.b. During this pregnancy, at any antenatal visit in the clinic, did you receive any information about breastfeeding your baby exclusively for at least 6 months? | 0=No 1=Yes 9=Don't know | Original |  |  |

*Bolded text indicates the origin of the presented question

**Supplementary Table 2.** Alignment between responses to questions about specific ANC visits in the cognitive and 6 month interviews and observed visits

|  | Cognitive study vs. observation  (*n=29*) | 6 month vs. observation  (*n=29*) | Cognitive study vs. 6 month  (*n=30*) |
| --- | --- | --- | --- |
| 4-month visit | 79.3% | 72.4% | 90.0% |
| 6-month visit | 69.0% | 75.9% | 93.3% |
| 8-month visit | 58.6% | 48.3% | 86.7% |
| 9-month visit | 75.9% | 75.9% | 100% |

Supplementary Figure 1. Lowess smoother analysis of the difference of observed ANC visits and cognitive recalled visits (y-axis) across total number of observed visits (x-axis).
